# Understanding the impact of spatial immunophenotypes on the survival of endometrial cancer patients through the ProMisE classification

**DOI:** 10.1007/s00262-024-03919-8

**Published:** 2025-01-03

**Authors:** Satomi Hattori, Nobuhisa Yoshikawa, Wenting Liu, Tetsuya Matsukawa, Mei Kubokawa, Kosuke Yoshida, Masato Yoshihara, Satoshi Tamauchi, Yoshiki Ikeda, Akira Yokoi, Yusuke Shimizu, Kaoru Niimi, Hiroaki Kajiyama

**Affiliations:** 1 Department of Obstetrics and Gynecology, Graduate School of Medicine, Nagoya University, 65, Tsurumai-Cho, Showa-Ku, Nagoya, Aichi 466-8560 Japan; 2 Department of Obstetrics and Gynecology Collaborative Research, Bell Research Center, Nagoya University Graduate School of Medicine, Nagoya, Japan

**Keywords:** Endometrial cancer, Tumor-infiltrating lymphocyte, ProMisE classification, Immunophenotype, Personalized immunotherapy

## Abstract

**Objectives:**

We focused on how the immunophenotypes based on the distribution of CD8-positive tumor-infiltrating lymphocytes (TILs) relate to the endometrial cancer (EC) molecular subtypes and patients’ prognosis.

**Patients and methods:**

Two cohorts of EC patients (total *n* = 145) were analyzed and categorized using the Molecular Risk Classifier for Endometrial cancer (ProMisE): POLEmut (*POLE* mutation), MMRd (mismatch repair deficiency), NSMP (no specific molecular profile), and p53abn (p53 abnormality). CD8-positive TILs, within the central tumor and the invasive margin, were examined by using immunohistochemical staining and advanced image-analysis software. It was investigated whether these immunophenotypes correlate with the molecular subtypes and patients' survival. RNA-sequencing (RNA-seq) was used to explore tumor-derived factors influencing these immunophenotypes.

**Results:**

Three distinct immunophenotypes (inflamed, excluded, and desert) based on the CD8-positive TIL patterns were identified in EC patients. Notably, the inflamed phenotype was most frequently observed in the POLEmut and MMRd subtypes, while the desert phenotype was predominant in the NSMP subtype; however, other immunophenotypes were also observed. All p53abn subtype showed the non-inflamed (excluded or desert) phenotype. The prognosis was markedly poorer in the patients with the non-inflamed phenotype than in those with the inflamed phenotype. The RNA-seq analysis showed that the expression of *MYC* target genes and type-1 interferon response genes was enriched in the non-inflamed phenotype in MMRd and NSMP subtypes, respectively.

**Conclusion:**

Evaluating not only the molecular classification but also the immunophenotype may lead to more personalized immunotherapy in EC and elucidating the mechanisms that underlie the formation of the three immunophenotypes could lead to the discovery of new immunotherapy targets.

**Supplementary Information:**

The online version contains supplementary material available at 10.1007/s00262-024-03919-8.

## Introduction

As of 2024, endometrial cancer (EC) ranks as the sixth most common malignancy in women globally, contributing significantly to cancer-related morbidity and mortality [1]. Despite recent advancements in the early detection of EC and its treatment modalities, a significant number of patients with EC continue to suffer from poor prognoses, particularly those with advanced-stage and high-grade disease [2, 3]. This persistent challenge underscores the limitations of current prognostic tools and therapies, thereby necessitating a more nuanced understanding of the molecular and immunological aspects of EC.

The Cancer Genome Atlas (TCGA) has been instrumental in identifying four key genomic subgroups in EC: *POLE* (ultramutated), microsatellite instability (MSI) (hypermutated), copy-number low (CN low) (endometrioid), and copy-number high (CN high) (serous-like) [4]. These findings have significantly advanced our approach to personalized EC treatments and prognoses. Notably, tumors with *POLE* mutations have shown better outcomes, even in high-grade cases, whereas the CN high subtype, particularly with *TP53* mutations, often indicates a poorer prognosis [5, 6]. This diversity in outcomes among the EC subgroups underscores the necessity for additional markers to refine the risk stratification and personalize treatments [4]. The proactive molecular risk classifier for endometrial cancer (ProMisE), based on TCGA's genomic subgroups, represents a significant step forward in this context [7, 8]. By integrating *POLE* exonuclease domain mutations and immunohistochemistry for mismatch repair proteins and TP53, the ProMisE categorizes EC into four distinct groups: POLE-EDM, MMR-D, p53wt, and p53abn [7, 8]. It extends TCGA's foundational work by providing comprehensive prognostic information, enhancing patient management within the International Federation of Gynecology and Obstetrics (FIGO) 2023 staging system [9, 10].

The distribution of tumor-infiltrating lymphocytes (TILs) was reported to be associated with the prognosis of patients with colorectal cancer in 2006 [11]. The concept of immunophenotypes (i.e., inflamed, excluded, and desert) based on the spatial distribution of CD8^+^ TILs, which recognized as vital players in modulating tumor progression and influencing responses to immunotherapies, was introduced in 2016 [12]. These immunophenotypes are known to affect the effectiveness of treatments, particularly treatment with immune checkpoint inhibitors (ICIs) in some solid tumors [12, 13]. In EC, the amount of CD8^+^ TILs has been reported to be associated with patients’ prognosis [14]; however, the association between the distribution of CD8^+^ TILs and clinical outcomes has not been investigated. Although ICIs are increasingly being applied clinically in the treatment of EC, research on the tumor immune microenvironment of EC is lacking.

We sought to bridge this knowledge gap by exploring the distribution and prognostic relevance of CD8^+^ TIL-based immunophenotypes in EC, stratified by ProMisE molecular classification. We hypothesized that each molecular subtype may be associated with a distinct immunophenotype, which could have significant implications for patient prognoses and treatment responses. Our goal is to integrate genomic and immunological data to provide a more holistic view of the EC landscape, potentially paving the way for novel, targeted immunotherapeutic strategies for EC.

## Patients and methods

### Study design and patient cohorts

#### Prospective cohort

A prospective analysis was conducted to examine the relationship between immunophenotype and molecular classification, as well as survival outcomes. The cases of 60 patients with EC treated during the period from January 2019 to December 2022 at Nagoya University Hospital (Nagoya, Japan) were prospectively enrolled. Following a definitive pathological diagnosis, these patients underwent surgical intervention. Ethical approval of this study was granted by the Institutional Review Board of Nagoya University (IRB approval no.: 2018-0400). Both fresh frozen and formalin-fixed paraffin-embedded (FFPE) tumor tissues were collected for analysis.

#### Retrospective cohort

We retrospectively analyzed the cases of a separate cohort of patients with high-grade EC (*n* = 85, including endometrioid grade 3, serous, and clear cell histology) treated between January 2002 and December 2017 at Nagoya University Hospital. FFPE tumor tissues were collected for analysis.

### Diagnosis and treatment

All 145 of the patients underwent a simple or semiradical hysterectomy with a bilateral salpingo-oophorectomy, pelvic, and/or para-aortic lymphadenectomy. The surgical specimens were staged according to the FIGO 2008 staging system. Postoperative treatment followed the Japan Society of Gynecologic Oncology guidelines [15, 16]. Risk-based adjuvant therapy was administered; low-risk patients received no additional treatment, and the intermediate- to high-risk patients received radiotherapy or platinum-based chemotherapy.

### Sanger sequencing for POLE exonuclease domain

Hotspot mutations in exons 9, 13, and 14 of the *POLE* gene were identified using Sanger sequencing. Genomic DNA extraction was conducted in accord with the manufacturers' protocols for both fresh frozen (NucleoSpin® DNA Rapidlyse, Macherey–Nagel, Düren, Germany) and FFPE tissues (QIAamp DNA FFPE Advanced UNG Kit, Qiagen, Hilden, Germany). The samples were enriched using the Blend Taq Plus (Toyobo, Osaka, Japan). The polymerase chain reaction (PCR) amplification and conditions were as described [17]. A portion of the PCR products was run on a 2% agarose gel in 1× TAE buffer to verify the presence of a single band approx. 200–300 base pairs in size. The rest of the PCR products was then purified using the QIAquick Gel Extraction Kit (Qiagen), following the manufacturer's instructions.

The DNA concentrations were measured with a NanoDrop One spectrophotometer (Thermo Fisher Scientific, Waltham, USA). The subsequent DNA sequencing was outsourced to Eurofins Genomics (Tokyo). For the sequence analysis, we used SnapGene Viewer ver. 6.1.2 (GSL Biotech, San Diego, CA) to examine the waveform patterns. Mutation identification was conducted using the Nucleotide BLAST tool (Basic Local Alignment Search Tool) from the U.S. National Center for Biotechnology Information (NCBI). In this study, we defined '*POLE* pathogenic variants' as the nine single-nucleotide substitutions on exons 9, 13, and 14: c.857 C > G (P286R) (exon9), c.884 T > G (M295R) (exon9), c.890 C > T (S297F) (exon9), c.1231 G > T/C (V411L) (exon13), c.1270 C > A (L424I) (exon13), c.1307 C > G (P436R) (exon13), c.1331 T > A (M444K) (exon13), c.1366 G > C (A456P) (exon14), and c.1376 C > T (S459F) (exon14) [18].

### Immunohistochemistry analysis

Immunohistochemistry (IHC) was conducted on 4-µm sections of FFPE tumor tissues, which included sections from both the central tumor (CT) and the invasive margin (IM). The primary antibodies used for the IHC included anti-human CD8 (clone C8/144b, Dako, Glostrup, Denmark; 1:100), PMS2 (clone A16-4, Biocare Medical, Walnut Creek, CA; 1:100), MSH6 (clone BC/44, Biocare Medical, 1:100), and p53 (clone DO-7, Dako; 1:100). The sections were deparaffinized and rehydrated, subjected to antigen retrieval in 10 mM sodium citrate (pH 6.0) or 1× Immunoactive (pH 9.0, Matsunami, Osaka, Japan) for 20 min at 95 °C in a microwave, and treated with 0.3% hydrogen peroxide in methanol for 20 min. Blocking was performed using the Histofine SAB-PO kit (Nichirei, Tokyo), followed by overnight incubation at 4 °C with the diluted primary antibodies. After the primary antibody incubation, the sections were incubated with biotin-labeled secondary antibody, peroxidase-labeled streptavidin, and developed using 3,3'-diaminobenzidine (DAB) substrate-chromogen for specific time durations. Then, the sections were counterstained with hematoxylin, dehydrated, and mounted.

Our application of immunohistochemistry for PMS2 and MSH6 was based on reports suggesting their effectiveness in screening for mismatch repair deficiency (MMRd) [19]. MMRd was identified by the complete absence of nuclear staining for either protein with internal positive controls including unaltered nuclear staining in adjacent normal endometrium, stromal cells, and inflammatory cells. Representative examples of loss of MSH6 or PMS2 expression are shown in Supplementary Fig. S1A–D. Abnormal p53 staining (p53abn) was characterized as either a strong, diffuse nuclear staining pattern in > 80% of carcinoma cells, or a complete lack of staining ("null pattern"), using adjacent non-tumor cells as an internal control. Wild-type tumor cells exhibited weak and heterogeneous staining patterns [20]. Representative examples of normal and abnormal p53 immunohistochemical staining patterns are shown in Supplementary Fig. S1E-G.

Multiplex immunofluorescent (IF) staining was performed by the TSA method using Opal IHC kit (PerkinElmer, Waltham, MA) according to the manufacture’s instructions. Anti-pan cytokeratin (clone AE1/AE3, Abcam, Cambridge, UK; 1:600) and anti-human CD8 (clone C8/144b, Dako, Glostrup, Denmark; 1:100) were used as primary antibodies. The antigen retrieval process was carried out as described above, and blocking was performed using the opal kit reagent, followed by incubation at room temperature for 30 min for anti-pan cytokeratin and overnight incubation at 4 °C for anti-CD8. After the primary antibody incubation, the sections were incubated with peroxidase-labeled secondary antibody, followed by incubation with opal 520 or opal 620 reagent. Then, the sections were stained with 4’, 6-diamidino-s-phenylindole (DAPI) (Dojindo, Kumamoto, Japan) and mounted. Multiplexed fluorescent labeled images were captured with a BZ-X800 (Keyence, Osaka, Japan).

### Immunophenotyping

The assessment of TILs in this study followed the guidelines established by the International Immuno-Oncology Biomarker Working Group [21]. We did not differentiate between intratumoral TILs and stromal TILs during this evaluation. After capturing stained slide images with a VS120-S5 (Evident, Tokyo, Japan), CD8^+^ TILs were quantified automatically in both the CT and IM using QuPath ver. 0.3.0 [22]. This quantification was performed over five distinct areas, each being a square with 0.25 mm on each side (0.0625 mm^2^ per square). The average number of CD8^+^ TILs per square millimeter was calculated for these regions.

The distinction between the three immunophenotypes (inflamed, excluded, and desert) was based on previous research [13, 23], but as there is no established definition for the density of CD8^+^ TILs in EC, we adopted 1000 cells/mm^2^ in this study. Tumors with a CD8^+^ TIL density ≥ 1000 cells/mm^2^ in both the CT and IM regions were classified as 'inflamed' phenotype. The tumors with a CD8^+^ TIL density < 1000 cells/mm^2^ in the CT but > 1000 cells/mm^2^ at the IM were designated as the 'excluded' phenotype. Conversely, tumors were classified as the 'desert' phenotype when the density of CD8^+^ TILs was < 1000 cells/mm^2^ in both the CT and IM areas.

### The ProMisE molecular classification

We conducted the molecular classification of tumors using an adapted approach from the ProMisE methodology, aligned with the steps outlined in the World Health Organization (WHO) classification [8]. This approach involved a sequential assessment of specific molecular markers. Initially, all tumor samples underwent Sanger sequencing to identify mutations in the *POLE* exonuclease domain, specifically targeting exons 9, 13, and 14 as noted above. Tumors harboring pathogenic variants in these regions were classified as 'POLEmut.' Next, the tumors exhibiting a complete absence of nuclear staining for PMS2 or MSH6 protein by IHC were categorized as 'MMRd.' Then, the tumors demonstrating abnormal p53 expression patterns by IHC were classified as 'p53abn.' Finally, tumors that did not exhibit any of the aforementioned molecular characteristics were classified as 'NSMP,' indicating a no specific molecular profile.

### Survival analysis

We defined progression-free survival (PFS) as the duration from the initiation of a patient's treatment to the point of observed disease progression. Overall survival (OS) was determined as the period from the commencement of treatment to either the death of a patient due to any reason or the patient's last confirmed survival status, with data collected up until March 2023 in retrospective cohort or until November 2024 in prospective cohort. We used the Kaplan–Meier method to estimate the 145 patients' PFS and OS rates. To assess the impact of immunophenotypes on the prognosis of EC patients within each molecular classification, survival curves were compared across the three immunophenotypes (inflamed, excluded, and desert) within each ProMisE subtype.

Given the limited sample size of our cohort, the abundance of CD8^+^ TILs from the Cancer Genome Atlas (TCGA) database was estimated using the CIBERSORT algorithm in R [24]. Patients in the TCGA database were stratified into high and low CD8^+^ T-cell groups within the four genomic subgroups of EC (POLE, MSI, CN low, and CN high) using a receiver operating characteristic (ROC) curve. An optimal cutoff value of 0.0536085 was adopted for predicting 1-year OS.

### RNA-sequencing analysis

The RNA-sequencing analysis was carried out on samples from the 40 of the 60 patients in prospective cohort from whom RNA samples were available. The extraction of RNA from fresh frozen tumor tissue was done using the NucleoSpin RNA Plus kit (Macherey–Nagel), following the manufacturer's guidelines. We measured the total RNA concentration with the NanoDrop One spectrophotometer. RNA-sequencing was then performed by Novogene Japan (Tokyo). The obtained raw FASTQ data were uploaded to Galaxy, an open-source web-based platform tailored for data-intensive biomedical research. Quality control of the data was executed using FastQC and Trimmomatic. The clean, paired-end data were then processed for gene expression quantification using the Kallisto quant tool, referencing the GENCODE GRC38.p13 transcript (genecode. v41.transcript).

Post-processing, the data were aggregated using the tximport package (ver. 1.18.0) in R software (ver. 4.0.3) and RStudio. For the subsequent analyses, scaled transcripts per million (TPM) counts were used. The TPM counts were processed with the use of the web portal for integrated differential expression and pathway analysis (iDEP) (iDEP 2.01; http://bioinformatics.sdstate.edu/idep, accessed April 30, 2024). We also used iDEP 2.01 for a principal component analysis (PCA). A gene set enrichment analysis (GSEA) was conducted employing the GSEA software (ver. 4.3.2), allowing for the identification of significantly altered pathways and gene sets in the dataset.

### Statistical analysis

We used GraphPad Prism software, ver. 9.2.0 (GraphPad Software, San Diego, CA) for the statistical analyses. To compare the relationships between different groups, we applied two distinct statistical tests depending on the data structure and distribution: the Wilcoxon matched-pairs signed-rank test was used for paired data comparisons, and the nonparametric Mann–Whitney U-test was applied for unpaired data sets. To compare distributions between the observed and expected data, we used the χ^2^-test.

The Kaplan–Meier method was used for the survival analyses, i.e., the PFS and OS rates. This allowed us to plot survival curves and estimate survival probabilities over time. The differences in survival rates between groups were evaluated by the log-rank test. Throughout the analyses, a p value threshold < 0.05 was set for determining statistical significance.

## Results

### The clinicopathological characteristics and ProMisE classification of prospective cohort

We conducted a prospective study over a 4-year period, enrolling consecutive 60 EC patients representing a full spectrum of histology. The clinicopathological characteristics of these patients are provided in Table 1. The cohort had a median follow-up of 38.4 months (range 0.6–66.0 months) and a median age of 58 years (range 32–84 years). Most of the patients (61.7%) presented with endometrioid G1/2 histology, and 66.7% were diagnosed at early stages (FIGO stage I–II). The ProMisE classification revealed that 11.7% of these patients fell into the POLEmut subtype, 25.0% into MMRd, 48.3% into NSMP, and 15.0% into p53abn (Table 2). The detailed *POLE* pathogenic variants are presented in Supplementary Table S1. The distribution of these categories aligns with another investigation of Japanese patients with EC [17]. Notably, higher ages were observed in the p53abn subtype, and the NSMP subtype predominantly consisted of endometrioid G1 and G2 histology. The serous histology tumors were exclusively categorized as p53abn.

**Table 1:** Prospective cohort; EC patients in 2019–2022

Total patients, *n*	60	
Follow–up period, mos.; median (range)	38.4 (0.6–66.0)	
Age, yrs; median (range)	58.0 (32–84)	
*Histology*		
Endometrioid G1/2	37	(61.7)
Endometrioid G3	20	(33.3)
Serous	3	(5.0)
*Stage (FIGO 2008)*		
I	35	(58.3)
II	5	(8.3)
III	18	(30.0)
IV	2	(3.3)
*Risk of recurrence*		
Low	13	(21.7)
Intermediate	18	(30.0)
High	29	(48.3)

The data are numbers and percentages

*EC* endometrial cancer; *FIGO* international federation of gynecology and obstetrics

**Table 2:** ProMisE molecular classification in prospective cohort

	POLEmut (*n* = 7)	MMRd (*n* = 15)	NSMP (*n* = 29)	p53abn (*n* = 9)
Proportion, %	11.7	25.0	48.3	15.0
Age, yrs; median	57.0	58.0	58.0	66.0
(range)	(48–75)	(50–65)	(32–84)	(52–75)
*Histology*				
Endometrioid G1/2	5	6	23	3
Endometrioid G3	2	9	6	3
Serous	0	0	0	3
*Stage (FIGO 2008)*				
I	6	7	19	3
II	0	1	4	0
III	1	6	6	5
IV	0	1	0	1
*Risk of recurrence*				
Low	2	2	8	1
Intermediate	4	4	10	0
High	1	9	11	8

*ProMisE* proactive molecular risk classifier for endometrial cancer; *POLEmut* polymerase-epsilon mutation; *MMRd* mismatch repair deficiency; *NSMP* no specific molecular profile; *p53abn* p53 abnormality; *FIGO* international federation of gynecology and obstetrics

### Three distinct immunophenotypes based on the distribution of CD8+ TILs

**Fig. 1:**
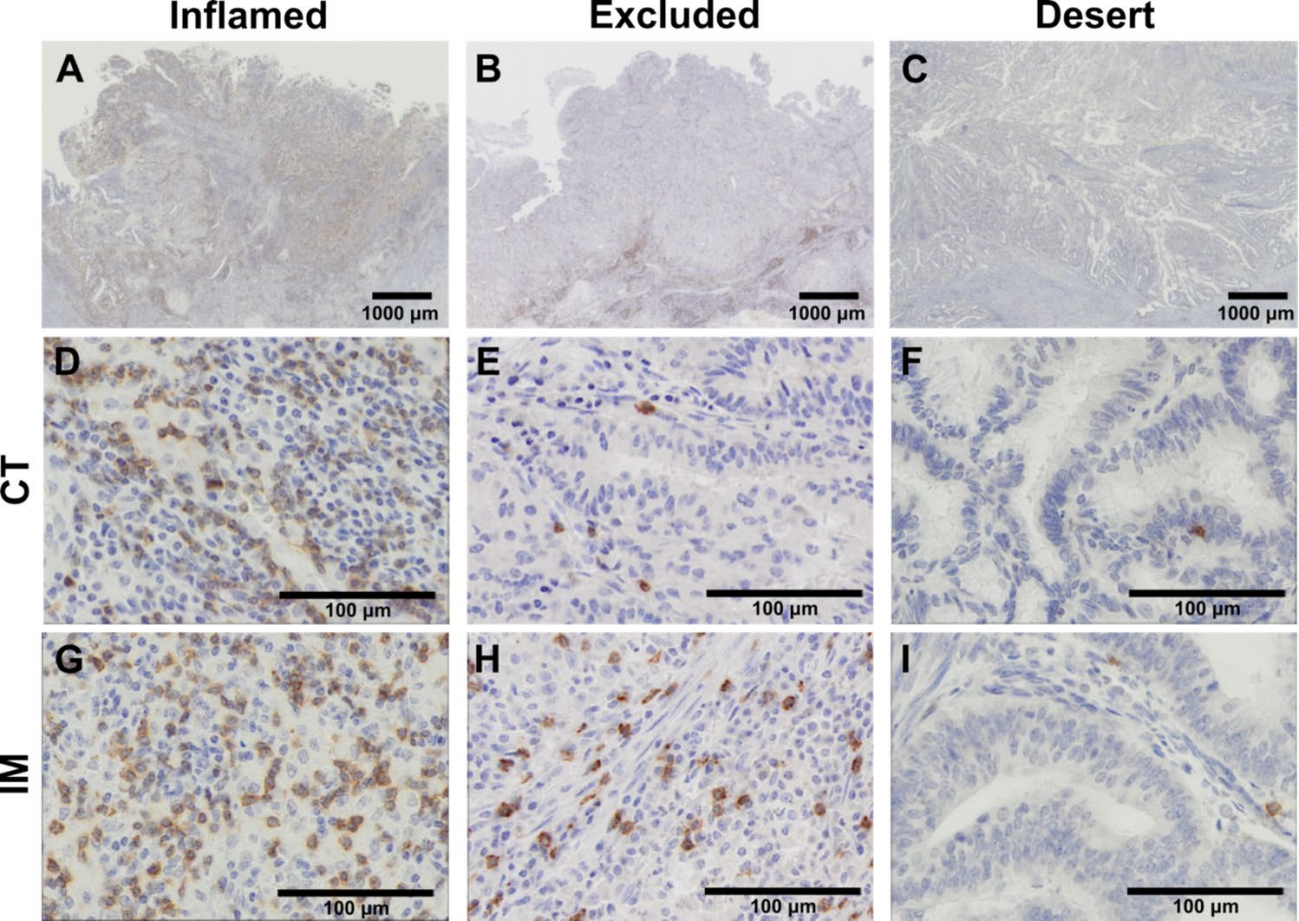
Representative images of three immunophenotypes based on the distribution patterns of CD8^+^ tumor-infiltrating lymphocytes (TILs) in endometrial cancer. The immunohistochemistry images of CD8^+^ TILs in the inflamed phenotype (**A**, **D**, **G**), excluded phenotype (**B**, **E**, **H**), and desert phenotype (**C**, **F**, **I**) are shown. The middle rows (**D**, **E**, **F**) show CD8^+^ TILs in the CT, and the bottom rows (**G**, **H**, **I**) show those in the IM. Abbreviations: CT, central tumor; IM, invasive margin

Figure 1 provides representative histological images of the three immunophenotypes; inflamed (Fig. 1A), excluded (Fig. 1B), and desert (Fig. 1C). The classification was based on the distribution patterns of CD8^+^ TILs. The inflamed phenotype showed abundant CD8^+^ TILs in both the CT (Fig. 1D) and the IM (Fig. 1G), whereas the excluded phenotype had a higher concentration in the IM (Fig. 1H) than the CT (Fig. 1E). The desert phenotype was characterized by sparse CD8^+^ TILs in both areas (Fig. 1F, I). To further evaluate the spatial relationship between tumor cells and CD8^+^ TILs, we performed multiplex IF staining for pan cytokeratin (tumor cell marker) and CD8. In the inflamed phenotype, CD8^+^ TILs were abundant in both the CT (Supplementary Fig. S2A, D) and the IM (Supplementary Fig. S2G, J), and CD8^+^ TILs well infiltrated into the tumor cells in both regions. In the excluded phenotype, there were many CD8^+^ TILs in the IM (Supplementary Fig. S2H, K), but few in the CT (Supplementary Fig. S2B, E). In the desert phenotype, there were few CD8^+^ TILs in both the CT (Supplementary Fig. S2C, F) and the IM (Supplementary Fig. S2I, L).

### The relationship between the ProMisE classification and the immunophenotypes in prospective EC cohort

**Fig. 2:**
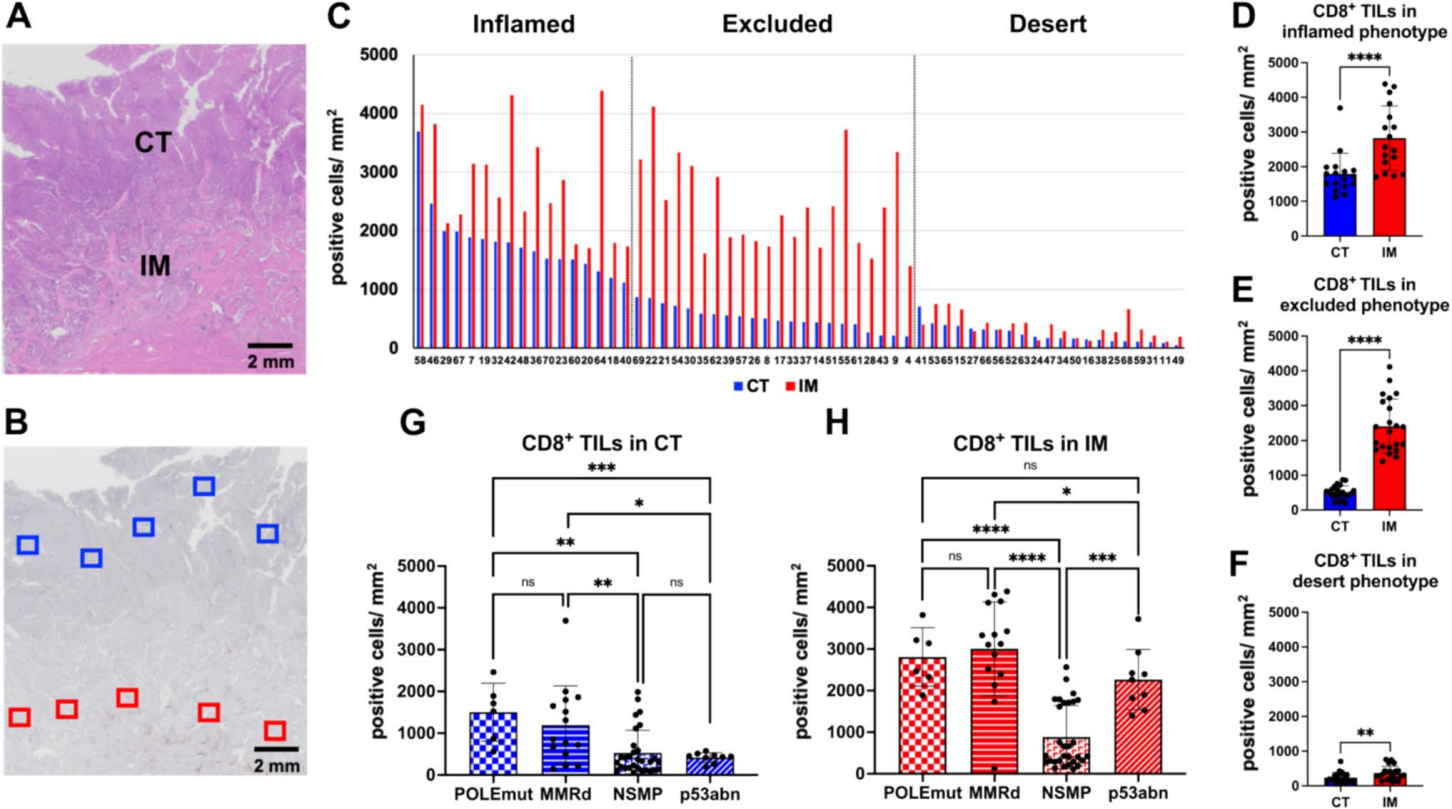
The relationship between the ProMisE classification and the immunophenotypes in prospective cohort. The approximate areas of the CT and IM in the hematoxylin and eosin stain are shown (**A**). CD8^+^ tumor-infiltrating lymphocytes (TILs) were automatically counted at five areas in the CT (*surrounded by blue lines*) and IM (*surrounded by red lines*) (**B**). The average density of CD8^+^ TILs (positive cells per square millimeter) in the CT (*blue bar*) and IM (*red bar*) for 60 EC patients is shown (**C**). Comparison of the density of the CD8^+^ TILs in the CT and IM in the inflamed phenotype (**D**), excluded phenotype (**E**), and desert phenotype (**F**). Comparison of the density of the CD8^+^ TILs by the four ProMisE subtypes in the CT (**G**) and IM (**H**). Abbreviations: CT, central tumor; IM, invasive margin; TILs, tumor-infiltrating lymphocytes; POLEmut, polymerase-epsilon mutation; MMRd, mismatch repair deficiency; NSMP, no specific molecular profile; p53abn, p53 abnormality

The CT and IM regions on the hematoxylin and eosin stain are shown in Fig. 2A. CD8^+^ TILs were automatically quantified five distinct areas in both the CT and IM, as illustrated in Fig. 2B. The average density of CD8^+^ TILs (positive cells per square millimeter) in the CT (blue bar) and IM (red bar) for 60 EC patients is shown in Fig. 2C. Among all 60 patients, 17 (28.3%) were classified as the inflamed phenotype, 22 (36.7%) as the excluded phenotype, and 21 (35%) as the desert phenotype. The median density of CD8^+^ TILs in the CT was significantly lower than that in the IM in the inflamed phenotype (1712/mm^2^ vs. 2563/mm^2^, *p* < 0.0001) (Fig. 2D), in the excluded phenotype (487/mm^2^ vs. 2330/mm^2^, *p* < 0.0001) (Fig. 2E), and in the desert phenotype (172/mm^2^ vs. 318/mm^2^, *p* = 0.0025) (Fig. 2F).

Figure 2G depicts the density of CD8^+^ TILs in the CT in each ProMisE category. The median density of CD8^+^ TILs in the CT was not significantly different between the POLEmut and MMRd subtypes (1522/mm^2^ vs. 855/mm^2^, *p* = 0.2666) or between the NSMP and p53abn subtypes (315/mm^2^ vs. 442/mm^2^, *p* = 0.2841). This value was significantly higher in the POLEmut subtype compared to the NSMP and p53abn subtypes (*p* = 0.0004 and *p* = 0.0003, respectively) and was significantly higher in the MMRd subtype versus the NSMP and p53abn subtypes (*p* = 0.0023 and *p* = 0.0148, respectively).

Figure 2H illustrates the density of CD8^+^ TILs in the IM in each of the ProMisE categories. The median density of CD8^+^ TILs in the IM was not significantly different between the POLEmut and MMRd subtypes (2469/mm^2^ vs. 3125/mm^2^; *p* = 0.3322) but was significantly higher in p53abn subtype than in NSMP subtype (2265/mm^2^ vs. 429/mm^2^; *p* = 0.0002) unlike the densities in the CT. In terms of the median density of CD8^+^ TILs in the CT and IM, the POLEmut and MMRd subtypes showed the inflamed phenotype with more CD8^+^ TILs in both areas; the NSMP subtype showed the desert phenotype with fewer CD8^+^ TILs in both areas; and the p53abn subtype showed the excluded phenotype with fewer CD8^+^ TILs in the CT and more in the IM. However, looking at individual cases, some of the POLEmut and MMRd subtypes showed the non-inflamed phenotypes, and some of the NSMP subtype showed the inflamed phenotype.

### The analysis of the relationship between the ProMisE classification and the immunophenotypes in prospective and retrospective EC cohort

To further investigate the relationship between the ProMisE classification and immunophenotype, we added that a retrospective cohort consists of 85 EC patients treated prior to 2017 to our analysis. These patients' clinicopathological characteristics are summarized in Supplementary Table S2. The median follow-up period was 75.8 months (range 0.7–173.3 months). Most of the retrospective cohort (70.6%) presented with endometrioid G3 histology; the other patients presented serous, clear, and mixed histology. The ProMisE classification revealed that 7.1% of the retrospective cohort were the POLEmut subtype, 22.3% had the MMRd subtype, 42.4% showed NSMP subtype, and 28.2% were classified as p53abn subtype (Supplementary Table S3). The detailed *POLE* pathogenic variants are presented in Supplementary Table S4.

Table 3 summarizes the distribution of the three immunophenotypes in each ProMisE category in prospective and retrospective cohort. Notably, the inflamed phenotype was most frequently observed in the POLEmut and MMRd subtypes, while the desert phenotype was predominant in the NSMP subtype; however, other immunophenotypes were also observed. No cases of the inflamed phenotype were observed in the p53abn subtype. The distribution of the three immunophenotypes in each cohort is shown in Supplementary Table S5 (prospective cohort) and S6 (retrospective cohort).

**Table 3:** Relationship between the ProMisE classifications and immunophenotypes in prospective and retrospective cohorts

	POLEmut (*n* = 13)	MMRd (*n* = 34)	NSMP (*n*= 65)	p53abn (*n* = 33)	*p* value
Inflamed	6	22	9	0	*p* < 0.0001
Excluded	6	9	13	23	
Desert	1	3	43	10	

*ProMisE* proactive molecular risk classifier for endometrial cancer; *POLEmut* polymerase-epsilon mutation; *MMRd* mismatch repair deficiency; *NSMP* no specific molecular profile; *p53abn* p53 abnormality; *FIGO* international federation of gynecology and obstetrics

### Survival analysis of the 145 EC patients stratified by the ProMisE classification and their immunophenotypes

**Fig. 3:**
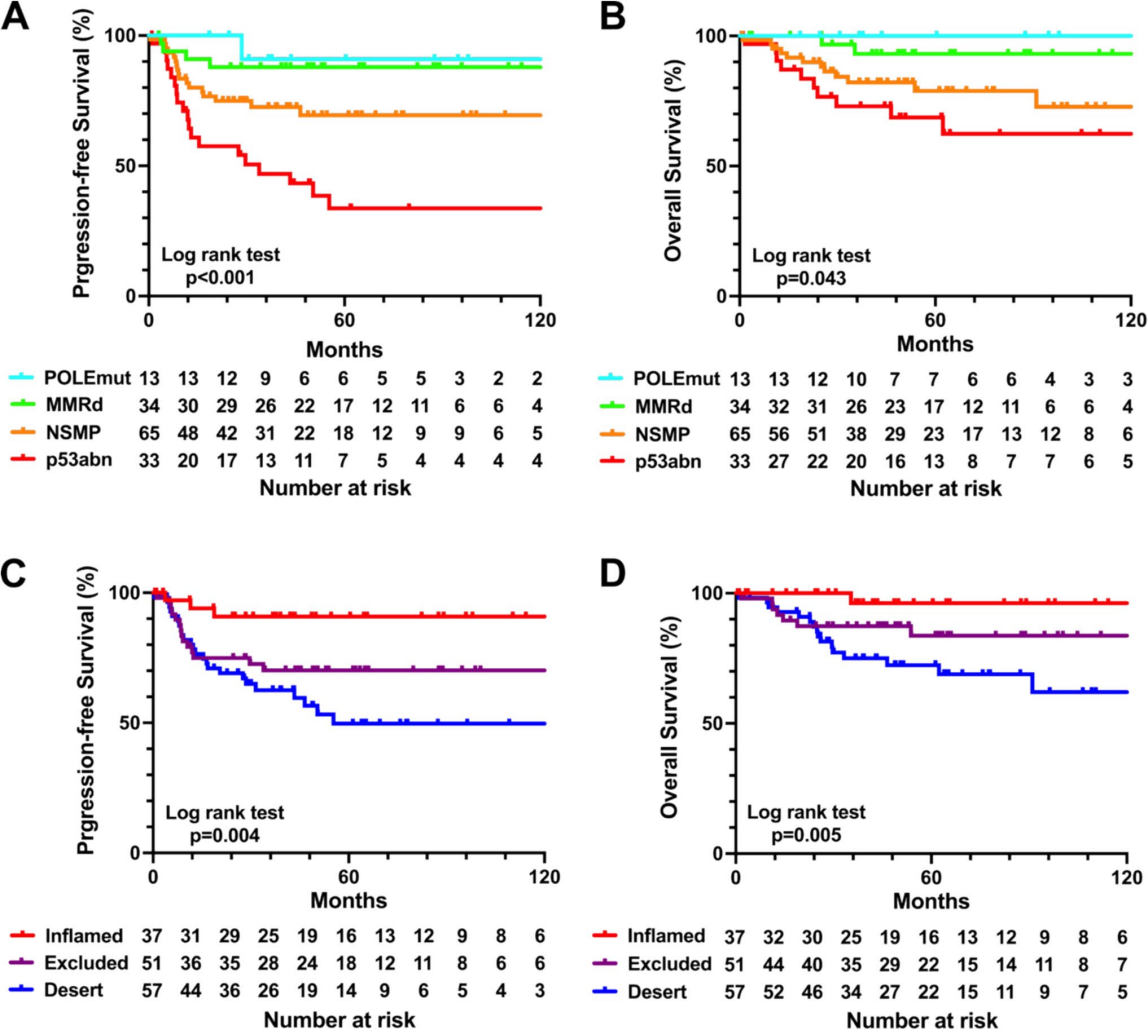
Survival analysis of 145 patients stratified by ProMisE classification, and the immunophenotypes. The progression-free survival and overall survival rates according to four ProMisE categories comprising POLEmut (*light blue line*), MMRd (*yellow-green line)*, NSMP (*orange line*), and p53abn (*red line*) subtypes are shown (**A**, **B**). The progression-free survival and overall survival rates according to three immunophenotypes comprising the inflamed (*red line*), excluded (*violet line*), and desert *(blue line*) phenotypes are shown (**C**, **D**). Abbreviations: ProMisE, proactive molecular risk cassifier for endometrial cancer; POLEmut, polymerase-epsilon mutation; MMRd, mismatch repair deficiency; NSMP, no specific molecular profile; p53abn, p53 abnormality

The survival analysis revealed distinct prognostic differences across the ProMisE classifications and immunophenotypes. The POLEmut and MMRd subtypes exhibited favorable PFS and OS rates, whereas the p53abn and NSMP subtypes were associated with poorer outcomes (Fig. 3A, B). Moreover, the patients with the excluded or desert phenotypes demonstrated significantly worse survival rates compared to those with the inflamed phenotype (Fig. 3C, D), highlighting the prognostic relevance of this immunophenotypic categorization. Within each ProMisE subtype, the prognostic trends of the immunophenotypes were generally maintained, with the inflamed phenotype associated with better outcomes compared to the excluded and desert phenotypes. However, due to the small number of patients in each subgroup, these trends did not reach statistical significance. Survival analysis by immunophenotype in each ProMisE subtype is depicted in Supplementary Figure S3.

### Survival analysis of the differences in the abundance of CD8+ T-cells in the four genomic subgroups of EC in the TCGA database

We utilized the TCGA database to verify whether the CD8^+^ T-cell fraction, estimated using the CIBERSORT algorithm, serves as a prognostic factor across the four molecular subtypes of EC in a larger cohort. Since the TCGA database does not provide direct immunophenotype data, we categorized cases based on the estimated CD8^+^ T-cell fraction. Cases with high CD8^+^ T-cell fractions were classified as CD8-high (analogous to the inflamed type), while those with low CD8^+^ T-cell fractions were grouped as CD8-low (representing non-inflamed types, including excluded and desert phenotypes). There were few cases in the CD8-low group in *POLE* subtype, and no differences in prognosis were observed between the two groups (Supplementary Fig. S4A, E). Although it was not significant, there was a trend for the prognosis of the CD8-low group to be worse than that of the CD8-high group in MSI (Supplementary Fig. S4B, F) and CN low (Supplementary Fig. S4C, G) subtypes. In the CN high subtype, the prognosis was significantly worse in the CD8-low group than in the CD8-high group (Supplementary Fig. S4D, H).

### Comparison of gene expression between the non-inflamed (excluded and desert) and inflamed phenotypes

**Fig. 4:**
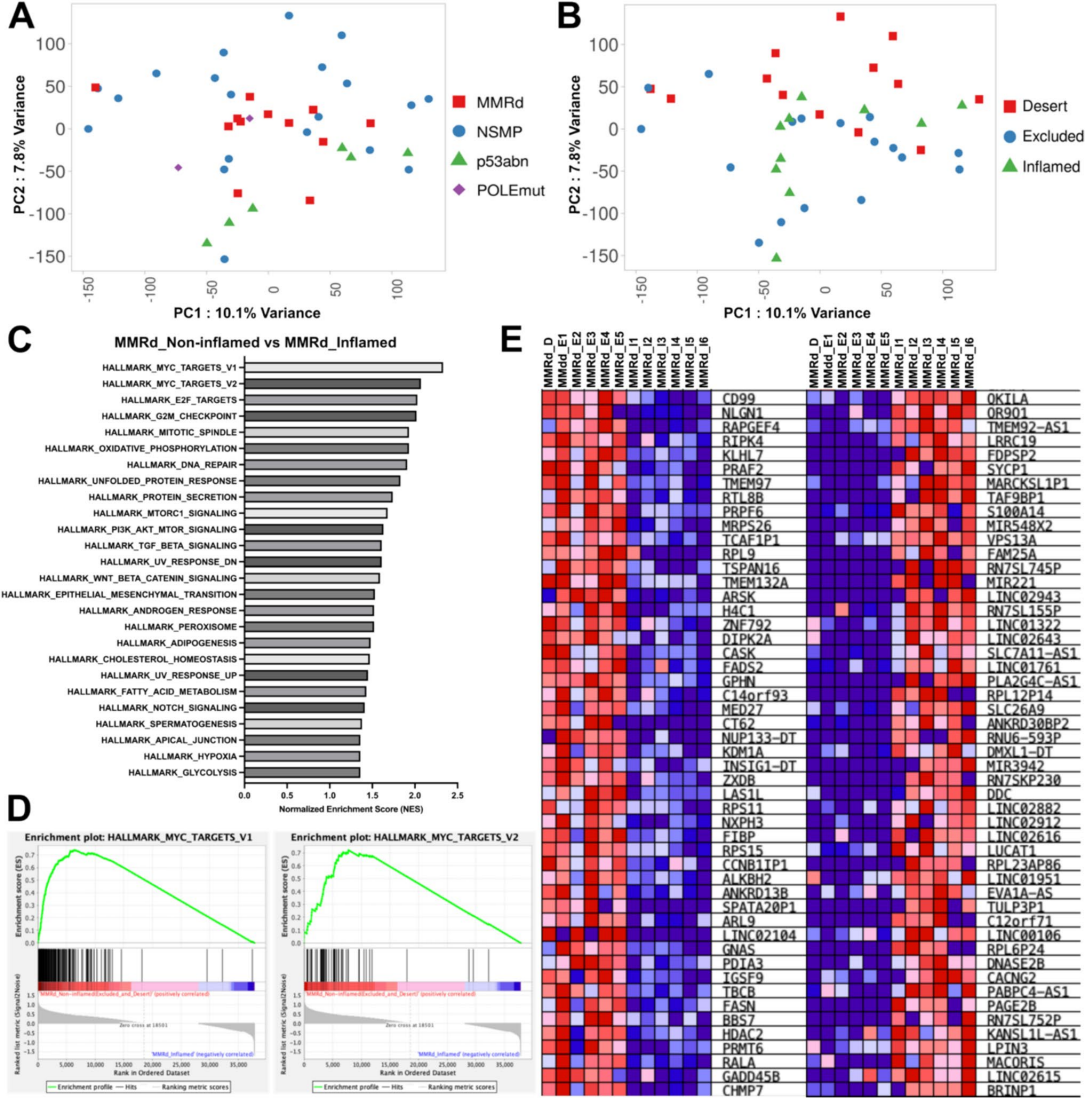
The results of the RNA-sequencing analysis in Cohort 2. Principal component analysis plots for the data of 40 samples in Cohort 2 are shown. The first two principal components are plotted and colored according to the ProMisE classification (**A**) or immunophenotype (**B**). The results of a gene set enrichment analysis (GSEA) of the MMRd subtype with the non-inflamed (excluded and desert) phenotypes versus that with the inflamed phenotype comparison are illustrated, as are the results of the GSEA hallmark analysis showing significantly enriched gene sets (FDR < 25% and a nominal *p* value < 5%). A positive normalized enrichment score indicates enrichment in the MMRd subtype with the non-inflamed phenotypes (**C**). Enrichment plots for the top two datasets enriched in the GSEA hallmark analysis, showing the profile of the running enrichment score and the positions of gene set members on the rank-ordered list (**D**). The heat map of the top 50 marker genes for each phenotype with the non-inflamed phenotypes (*left column*) versus that with the inflamed phenotype (*right column*). Expression values are represented as colors and range from *red* (high expression), *pink* (moderate), and *light blue* (low) to *dark blue* (lowest expression) (**E**). Abbreviations: PC, principal component; POLEmut, polymerase-epsilon mutation; MMRd, mismatch repair deficiency; NSMP, no specific molecular profile; p53abn, p53 abnormality

Finally, to investigate factors that produce the different distribution patterns of CD8^+^ TILs between the non-inflamed (excluded and desert) and inflamed phenotypes, we performed an RNA-sequencing (RNA-seq) analysis. The principal component analysis (PCA) was performed with RNA-seq data of 40 EC samples from prospective cohort. The first two principal components (PCs) are plotted and colored according to the ProMisE classification (Fig. 4A) or immunophenotype (Fig. 4B). The GSEA of the MMRd subtype with the non-inflamed phenotypes compared to that with the inflamed phenotype revealed that the expressions of *MYC* target gene sets were more enriched in the non-inflamed phenotypes versus the inflamed phenotype (Fig. 4C). Figure 4D shows the enrichment plots for the top two datasets that were enriched in the GSEA hallmark analysis. The heat map of the top 50 marker genes for each phenotype in the MMRd subtype with the non-inflamed phenotypes and that with the inflamed phenotype is provided as Fig. 4E. The top two genes that were upregulated in the non-inflamed phenotype were *CD99* and *NLGN1*. The top gene that was upregulated in the inflamed phenotype was *BRINP1.*

**Fig. 5:**
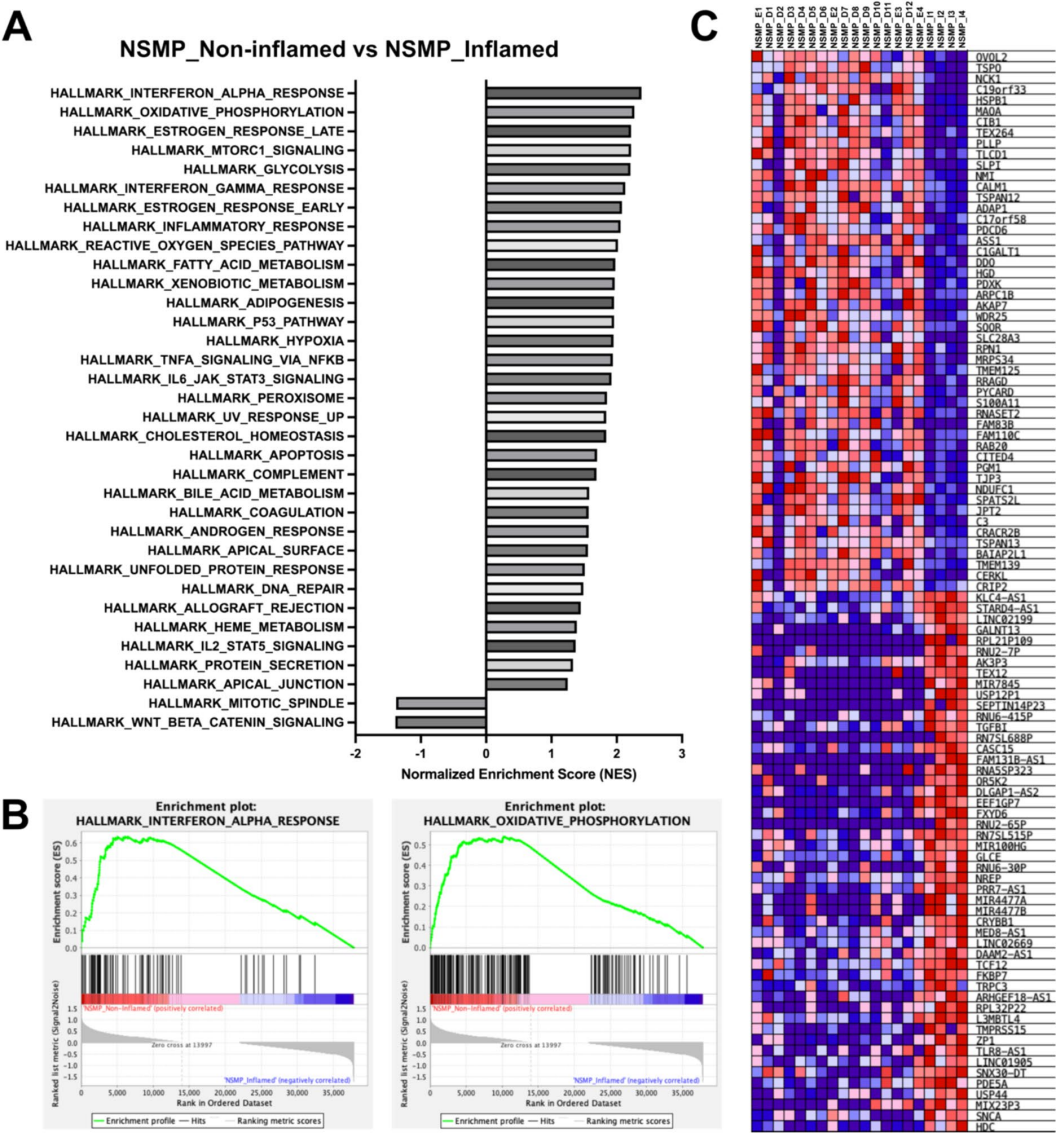
Gene set enrichment analysis (GSEA) results of the NSMP subtype with the non-inflamed (excluded or desert) phenotypes versus that with the inflamed phenotype in Cohort 2. The results of the GSEA hallmark analysis showing significantly enriched gene sets (FDR < 25% and a nominal *p* value < 5%). A positive normalized enrichment score indicates enrichment in the NSMP subtype with the non-inflamed phenotypes, and a negative score indicates enrichment in that with the inflamed phenotype (**A**). Enrichment plots for the top two datasets enriched in GSEA hallmark analysis, showing the profile of the running enrichment score and the positions of gene set members on the rank-ordered list (**B**). Heat map of the top 50 marker genes for each phenotype with the non-inflamed phenotypes (*left column*) versus that with the inflamed phenotype (*right column*). Expression values are represented as colors and range from *red* (high expression), *pink* (moderate), and *light blue* (low) to *dark blue* (lowest expression) (**C**). Abbreviation: NSMP, no specific molecular profile

Our GSEA of the NSMP subtype with the non-inflamed phenotypes compared to that with the inflamed phenotype revealed that the expressions of type-1 interferon response gene sets were more enriched in the non-inflamed phenotypes versus the inflamed phenotype (Fig. 5A). The enrichment plots for the top two datasets enriched in the GSEA hallmark analysis are given in Fig. 5B. The heat map of the top 50 marker genes for each phenotype in the NSMP subtype with the non-inflamed phenotypes and that with the inflamed phenotype is shown in Fig. 5C. The top gene that was upregulated in the non-inflamed phenotype was *OVOL2*. The top gene that was upregulated in the inflamed phenotype was *HDC.*

## Discussion

Our investigation into the distribution and prognostic significance of CD8^+^ TILs in EC according to a molecular classification revealed crucial findings. Most notably, we identified three distinct immunophenotypes—inflamed, excluded, and desert—based on CD8^+^TILs in EC patients. These immunophenotypes provide a more granular understanding of the immune landscape in EC, reflecting the diverse immunological responses triggered by tumor development. Our results demonstrated that inflamed phenotypes were associated with better prognosis, while excluded and desert phenotypes correlated with poorer outcomes. Importantly, the integration of immunophenotypes with the ProMisE molecular classification underscores the complexity of EC and highlights the need for personalized therapeutic strategies that consider both molecular and immunological characteristics.

Interestingly, we observed that the EC patients with the excluded or desert phenotypes had poorer prognoses than those exhibiting inflamed phenotypes. This observation aligns with the increasing body of evidence suggesting that a robust anti-tumor immune response, represented by a high level of CD8^+^ TILs, is associated with better outcomes in various cancers, including EC [14]. Additionally, TCGA-based analysis using the CIBERSORT algorithm supported this observation, revealing that cases with high CD8^+^ T-cell fractions (analogous to inflamed phenotypes) demonstrated better prognoses. These results emphasize the value of incorporating immunophenotypic evaluation, alongside molecular classification, into prognostic assessments for EC.

When evaluated by the median density of CD8^+^ TILs in the CT and IM, the POLEmut and MMRd subtypes showed the inflamed phenotype; the NSMP subtype showed the desert phenotype; and the p53abn subtype showed the excluded phenotype. This insight suggests a potential correlation between the genomic background and CD8^+^ T-cell anti-tumor response in EC. It has been reported that POLEmut and MMRd subtypes have high tumor mutation burdens which contribute to the expression of neoantigens and they cause a strong anti-tumor response by CD8^+^ T-cell [25]. The limited CD8^+^ T-cell response in the NSMP subtype suggests that this subtype is less likely to elicit a CD8^+^ T-cell response due to its low immunogenicity and may contribute to its resistance to immunotherapy strategies. Our observation that the p53abn subtype exhibited the excluded phenotype suggests that this subtype, despite eliciting an immune response, is resistant to immunotherapy because it has mechanisms that exclude CD8^+^ TILs from the central tumor. However, looking at individual cases, some of the POLEmut and MMRd subtypes showed the non-inflamed phenotypes, and some of the NSMP subtype showed the inflamed phenotype. It is thought that the formation of different immunophenotypes is due to some factors other than the number of genetic mutations or differences in immunogenicity.

Our RNA-seq analysis provided valuable insights into the molecular mechanisms underlying the formation of inflamed and non-inflamed phenotypes within the ProMisE subtypes. In the MMRd subtype, non-inflamed phenotypes exhibited upregulation of CD99 and NLGN1, both of which have been implicated in immune suppression and tumor progression. There have been reports that high expression of CD99 in tumors is involved in the infiltration of immunosuppressive macrophages, in addition to the malignant transformation of tumor cells themselves [26, 27], and some study reported that high expression of NLGN1 was a poor prognostic factor in colorectal cancer, prostate cancer, and pancreatic cancer [28, 29]. In contrast, inflamed phenotypes showed upregulation of BRINP1, a gene associated with immune cell differentiation and PD-L1 regulation in tumor cells, suggesting its role in fostering robust anti-tumor immune responses [30, 31]. In the NSMP subtype, the non-inflamed phenotypes were characterized by elevated expression of OVOL2, a potential tumor suppressor gene [32, 33], However, the role of OVOL2 in tumor immunity remains unclear. In contrast, inflamed phenotypes showed increased expression of HDC, an enzyme involved in histamine production, which has recently been associated with immune modulation in the tumor microenvironment [34, 35]. There is scope for further exploration of the impact of these genes on tumor immunity.

Our RNA-seq analysis also provided another layer of insight, suggesting that *MYC* target genes or type-1 interferon response genes might play a role in determining these immunophenotypes. The *MYC* oncogene is a well-documented driver of tumorigenesis in many cancers, and the type-1 interferon pathway is an important pathway in the antiviral response; however, their roles in the formation of the tumor immunosuppressive microenvironment remain unclear. Our findings suggest that the *MYC* signaling pathway or type-1 interferon pathway may, directly or indirectly, influence the distribution of CD8^+^ TILs and form a different immunophenotype, representing a novel avenue for future research.

There are some limitations to our research. First, we only assessed CD8^+^ TILs in the evaluation of the immune microenvironment of endometrial cancer, To clarify the immune microenvironment, it is necessary to assess the distribution patterns of immune cells other than CD8^+^ T-cells and the interaction of each immune cell and also necessary to assess the expression of immune evasion molecules such as PD-L1 expression on tumor cells, which is a future task. Next, there is a lack of functional analysis of the *MYC* target genes and type-1 IFN genes in terms of their effects on immune phenotypes and EC patients’ clinical outcomes, which is another topic for future research. Furthermore, because the number of tumors in which RNA-seq was performed was limited, it was not possible to find the factors that cause the differences between the excluded and desert phenotypes. As it is predicted that it will be difficult to find the differences in these two non-inflamed phenotypes using RNA-seq of only one region in the CT, we believe that it will be necessary to compare the gene expression in the CT and the IM in future research.

In conclusion, our results suggest that evaluating not only the molecular classification but also the immunophenotype may lead to more accurate patients’ prognosis prediction in EC. Future studies exploring the role of the *MYC* signaling pathway or type-1 interferon pathway in shaping the immune landscape will undoubtedly provide further insights into the complex biology of EC. Elucidating the mechanisms that underlie the formation of the three immunophenotypes could lead to the discovery of novel immunotherapy targets.

## Supplementary Information

Below is the link to the electronic supplementary material.

Supplementary file 1 (PDF 9661 KB)

Supplementary file 2 (PDF 41 KB)

## Data Availability

The datasets analyzed during the current study are available from the corresponding author on reasonable request.
