# Supplementary material for: Understanding the impact of spatial immunophenotypes on the survival of endometrial cancer patients through the ProMisE classification
Source: Cancer Immunol Immunother. 2025 Jan 3;74(2):70. doi: 10.1007/s00262-024-03919-8 (PMC11699169; doi:10.1007/s00262-024-03919-8)
Supplement: Supplementary file 1 — (PDF 9661 KB) [file 262_2024_3919_MOESM1_ESM.pdf]

**Suppl. Fig. S1.**

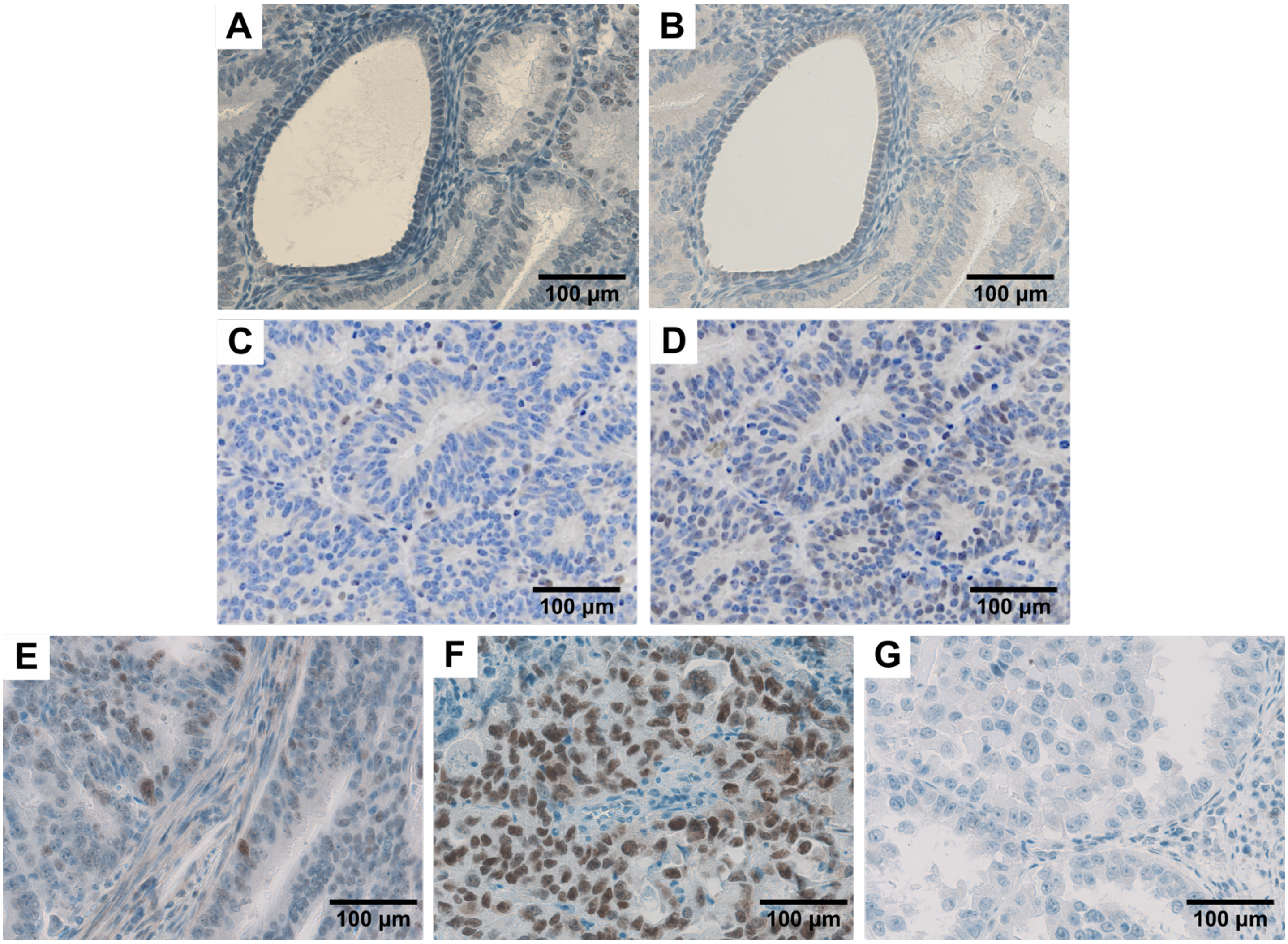


**Suppl. Fig. S1.** Representative images of PMS2, MSH6 and p53 immunohistochemical staining patterns.

PMS2-retained **(A)** and MSH6-lost **(B)** patterns in a patient. PMS2-lost **(C)** and MSH6-retained **(D)** patterns in another patient.

Normal p53 staining pattern **(E)** and abnormal p53 staining patterns; mutant overexpression **(F)** and null mutant **(G)**.

**Suppl. Fig. S2.**

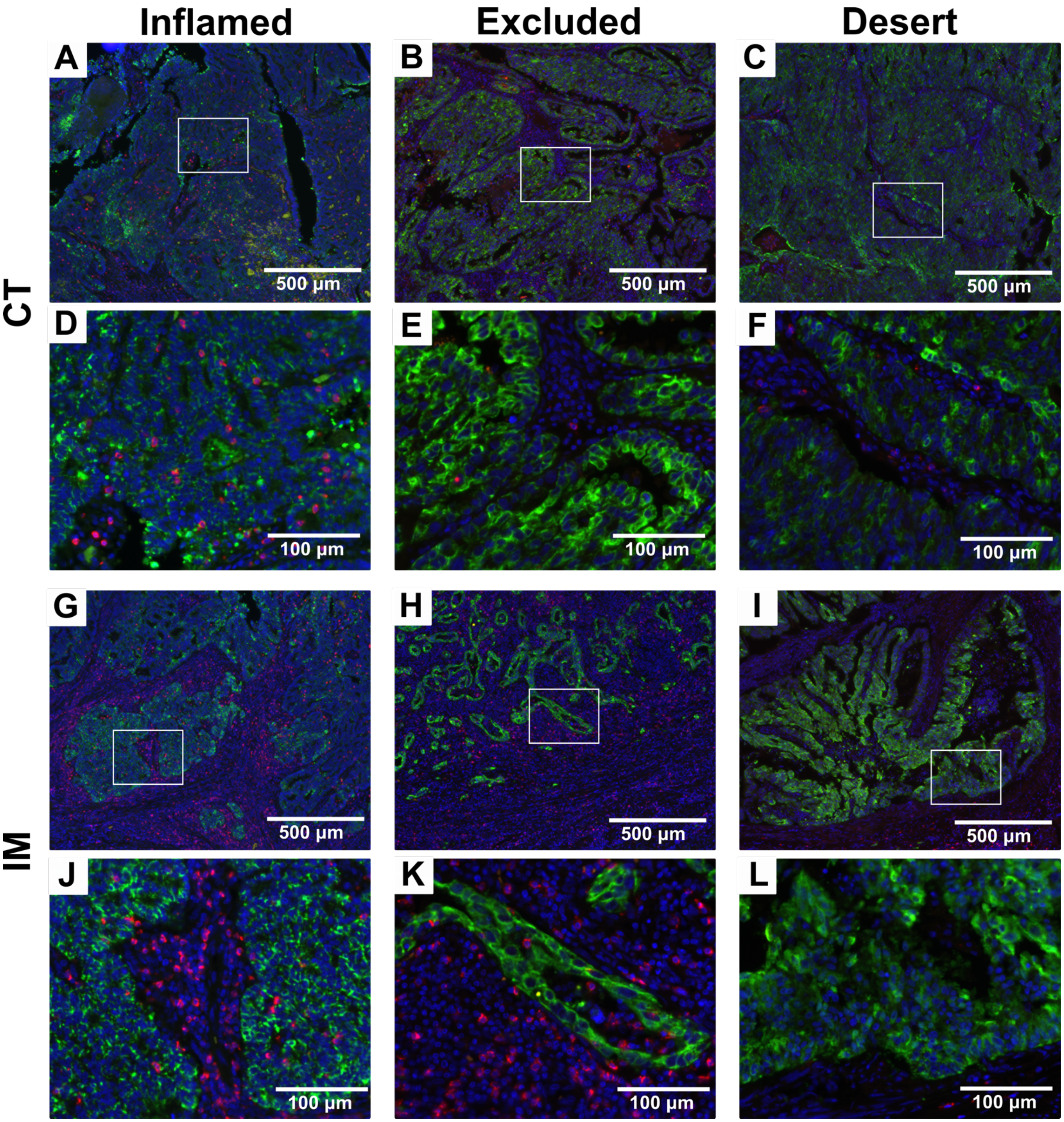


**Suppl. Fig. S2.** Representative multiplex immunofluorescent images of three immunophenotypes based on the distribution patterns of CD8$^{+}$ tumor-infiltrating lymphocytes (TILs) in endometrial cancer.

The multiplex immunofluorescent images of tumor cells (pan-cytokeratin, green), CD8$^{+}$ TILs (CD8, red) and DAPI (blue) in the CT **(A, D)** and in the IM **(G, J)** in the inflamed phenotype, in the CT **(B, E)** and in the IM **(H, K)** in excluded phenotype, and in the CT **(C, F)** and in the IM **(I, L)** in the desert phenotype **(C, F, I)** are shown.

Abbreviations: CT, central tumor; IM, invasive margin

**Suppl. Fig. S3.**

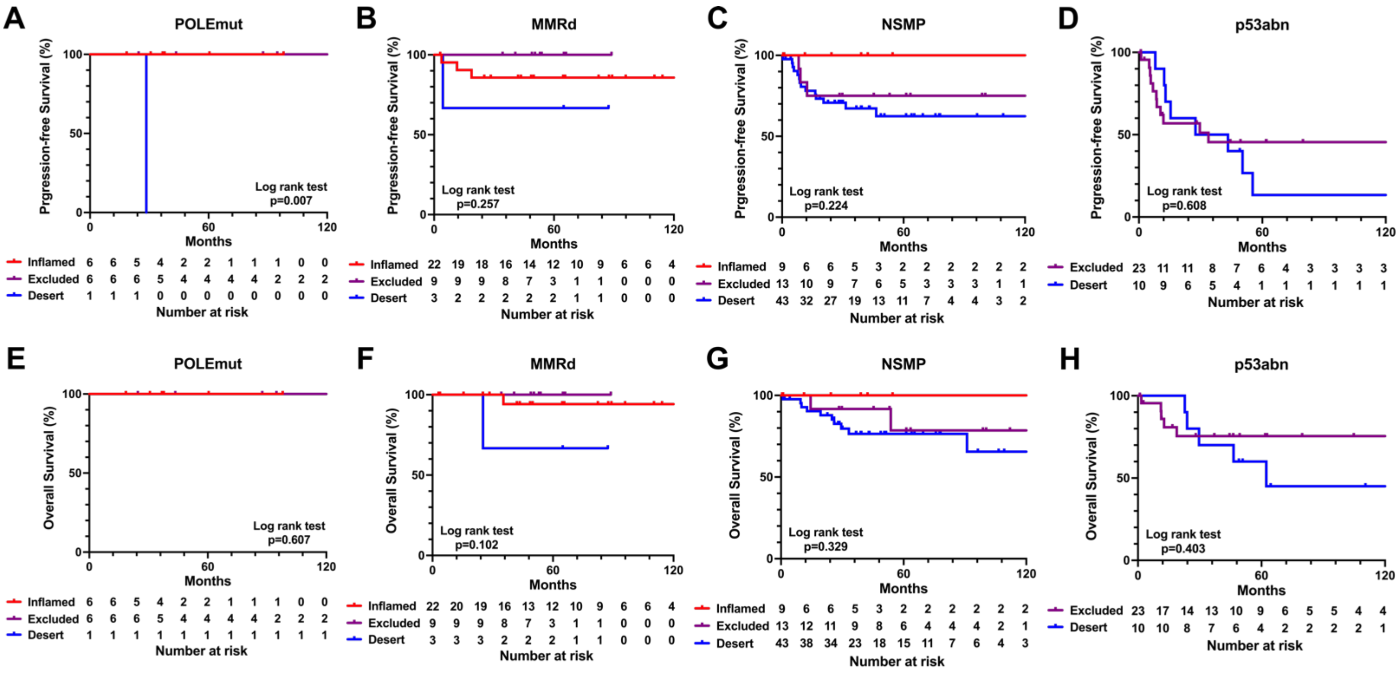


**Suppl. Fig. S3.** Survival analysis by immunophenotype in each Proactive Molecular Risk Classifier for Endometrial cancer (ProMisE) subtype.

The progression-free survival and overall survival rates according to immunophenotype in POLEmut **(A, E)**, MMRd **(B, F)**, NSMP **(C, G)**, and p53abn **(D, H)** subtypes.

Abbreviations: POLEmut, polymerase-epsilon mutation; MMRd, mismatch-repair deficiency; NSMP, no specific molecular profile; p53abn, p53 abnormality

**Suppl. Fig. S4**

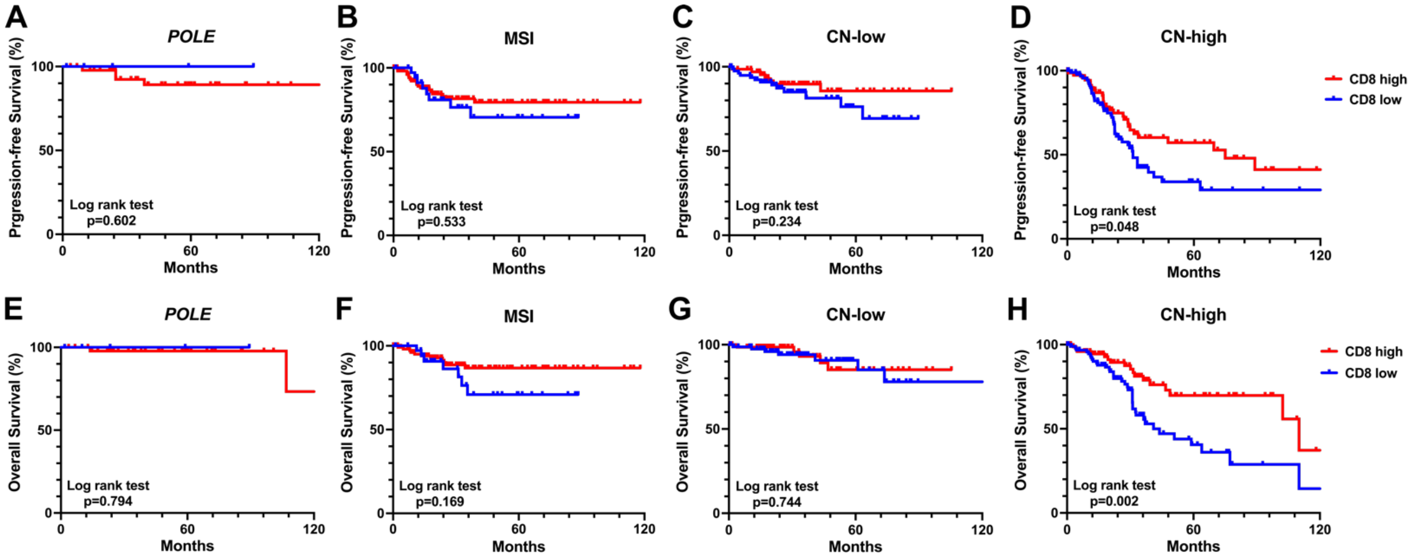


**Suppl. Fig. S4.** Survival analysis of the differences in the abundance of $CD8^+$ T cells in the four genomic subgroups of endometrial cancer in the Cancer Genome Atlas (TCGA) database.

The progression-free survival and overall survival rates by high or low levels of the $CD8^+$ T cells in *POLE* **(A, E)**, MSI **(B, F)**, CN-low **(C, G)**, and CN-high **(D, H)** subtypes.

Abbreviations: *POLE*, polymerase-epsilon; MSI, microsatellite instability; CN-low, copy-number low; CN-high, copy-number high
