# Supplementary material for: Understanding the impact of spatial immunophenotypes on the survival of endometrial cancer patients through the ProMisE classification
Source: Cancer Immunol Immunother. 2025 Jan 3;74(2):70. doi: 10.1007/s00262-024-03919-8 (PMC11699169; doi:10.1007/s00262-024-03919-8)
Supplement: Supplementary file 2 — (PDF 41 KB) [file 262_2024_3919_MOESM2_ESM.pdf]

Supplementary Table S1. *POLE* pathogenic variants identified in prospective cohort

**Nucleotide substitution**	**n**
Exon 9	
c.857 C>G	2
Exon 13	
c.1231 G>T	2
Exon 14	
c.1366 G>C	1
c.1376 C>T	2

Supplementary Table S2. Retrospective cohort; EC patients with high-grade histology in 2002–2017

Total patients, n	85	
Follow-up period, months; median (range)	75.8 (0.7–173.3)	
Age, yrs; median (range)	61.0 (36–84)	
Histology:		
Endometrioid G3	60	(70.6)
Serous	12	(14.1)
Clear	8	(9.4)
Endometrioid G3+Serous	1	(1.3)
Endometrioid G3+Clear	3	(3.5)
Others	1	(1.3)
Stage (FIGO 2008):		
I	35	(41.2)
II	10	(11.8)
III	27	(31.8)
IV	13	(15.3)
Risk of recurrence:		
Intermediate	13	(15.3)
High	72	(84.7)

The data are numbers and percentages.
Abbreviations: EC, endometrial cancer; FIGO, International Federation of Gynecology and Obstetrics

Supplementary Table S3. ProMisE molecular classification in retrospective cohort

	**POLEmut (n=6)**	**MMRd (n=19)**	**NSMP (n=36)**	**p53abn (n=24)**
Proportion, %	7.1	22.3	42.4	28.2
Age, yrs; median (range)	57.5 (36–73)	56.0 (43–84)	57.5 (39–82)	69.0 (38–82)
Histology:				
Endometrioid G3	5	15	31	9
Serous	1	1	0	10
Clear	0	3	2	3
Endometrioid G3+Serous	0	0	0	1
Endometrioid G3+Clear	0	0	2	1
Others	0	0	1	0
Stage (FIGO 2008):				
I	4	9	13	9
II	0	4	4	2
III	1	4	14	8
IV	1	2	5	5
Risk of recurrence:				
Intermediate	2	4	5	2
High	4	15	31	22

Abbreviations: ProMisE, Proactive Molecular Risk Classifier for Endometrial cancer; POLEmut, polymerase-epsilon mutation; MMRd, mismatch-repair deficiency; NSMP, no specific molecular profile; p53abn, p53 abnormality; FIGO, International Federation of Gynecology and Obstetrics

Supplementary Table S4. *POLE* pathogenic variants identified in retrospective cohort

Nucleotide substitution	n
Exon 9	
c.857 C>G	4
c.890 C>T	1
Exon 14	
c.1376 C>T	1

Supplementary Table S5. Relationship between the ProMisE classifications and immunophenotypes in prospective cohort

	POLEmut (n=7)	MMRd (n=15)	NSMP (n=29)	p53abn (n=9)	p value
Inflamed	5	7	5	0	
Excluded	2	7	4	9	p<0.0001
Desert	0	1	20	0	

Abbreviations: ProMisE, Proactive Molecular Risk Classifier for Endometrial cancer; POLEmut, polymerase-epsilon mutation; MMRd, mismatch-repair deficiency; NSMP, no specific molecular profile; p53abn, p53 abnormality; FIGO, International Federation of Gynecology and Obstetrics

Supplementary Table S6. Relationship between the ProMisE classifications and immunophenotypes in retrospective cohort

	POLEmut (n=6)	MMRd (n=19)	NSMP (n=36)	p53abn (n=24)	p value
Inflamed	1	15	4	0	
Excluded	4	2	9	14	p<0.0001
Desert	1	2	23	10	

Abbreviations: ProMisE, Proactive Molecular Risk Classifier for Endometrial cancer; POLEmut, polymerase-epsilon mutation; MMRd, mismatch-repair deficiency; NSMP, no specific molecular profile; p53abn, p53 abnormality; FIGO, International Federation of Gynecology and Obstetrics
